# Scientific workflow optimization for improved peptide and protein identification

**DOI:** 10.1186/s12859-015-0714-x

**Published:** 2015-09-03

**Authors:** Sonja Holl, Yassene Mohammed, Olav Zimmermann, Magnus Palmblad

**Affiliations:** Jülich Supercomputing Centre (JSC), Forschungszentrum Jülich, 52425 Jülich, Germany; Center for Proteomics and Metabolomics, Leiden University Medical Center, PO Box 9600, 2300 RC Leiden, The Netherlands

**Keywords:** Taverna workbench, Optimization, Scientific workflow, Tandem mass spectrometry, X!Tandem

## Abstract

**Background:**

Peptide-spectrum matching is a common step in most data processing workflows for mass spectrometry-based proteomics. Many algorithms and software packages, both free and commercial, have been developed to address this task. However, these algorithms typically require the user to select instrument- and sample-dependent parameters, such as mass measurement error tolerances and number of missed enzymatic cleavages. In order to select the best algorithm and parameter set for a particular dataset, in-depth knowledge about the data as well as the algorithms themselves is needed. Most researchers therefore tend to use default parameters, which are not necessarily optimal.

**Results:**

We have applied a new optimization framework for the Taverna scientific workflow management system (http://ms-utils.org/Taverna_Optimization.pdf) to find the best combination of parameters for a given scientific workflow to perform peptide-spectrum matching. The optimizations themselves are non-trivial, as demonstrated by several phenomena that can be observed when allowing for larger mass measurement errors in sequence database searches. On-the-fly parameter optimization embedded in scientific workflow management systems enables experts and non-experts alike to extract the maximum amount of information from the data. The same workflows could be used for exploring the parameter space and compare algorithms, not only for peptide-spectrum matching, but also for other tasks, such as retention time prediction.

**Conclusion:**

Using the optimization framework, we were able to learn about how the data was acquired as well as the explored algorithms. We observed a phenomenon identifying many ammonia-loss b-ion spectra as peptides with N-terminal pyroglutamate and a large precursor mass measurement error. These insights could only be gained with the extension of the common range for the mass measurement error tolerance parameters explored by the optimization framework.

## Background

In mass spectrometry based proteomics, whether bottom-up, top-down, or middle-down [1], the matching of a single tandem mass spectrum, or a spectral tree [2] to a peptide is an integral part of most methods for identifying peptides and proteins. Existing methods fall into one of three broad categories: sequence database searches [3], spectral libraries [4–6] and *de novo* sequencing [7]. Most recent methods can be applied to data from collision-induced dissociation [8], electron capture dissociation [9] or other fragmentation techniques, individually or in combination [10, 11]. The identification may be based on MS^2^, MS^3^ or a combination of these. Several groups have also published efforts in combining multiple algorithms for peptide-spectrum matching, for instance the framework developed by Searle et al. [12], the MSblender software from Kwon et al. [13] or the FDRAnalysis algorithm of Wedge et al. [14]. Recently, in de Bruin *et al*. [15] and Mohammed *et al*. [16] we have shown how some of these algorithms can be integrated with other algorithms in scientific workflows [17]. Scientific workflows enable researchers to concentrate on their research purpose rather than on computational challenges. However, all these algorithms use a number of user-defined input parameters, such as the specificity and fidelity of the enzymatic digestion, the sequences or library to search spectra against, mass measurement uncertainty or error (MME) and score or probability thresholds in the assembly of peptide-spectrum matches to peptide or protein sets. Typically, the choice of algorithm and parameters is determined from the users’ experience and expert knowledge about the experiment, instrumentation and data quality. Previously, Piehowski *et al*. have used a “systematic trial-and-error parameter selection” to optimize peptide identification using SEQUEST [18], showing significant improvement over using default search parameters. Here we describe the usage of a framework [19] for automated optimization of scientific workflows with two very different analysis tasks: peptide-spectrum matching and chromatographic retention time prediction. The optimization process can be reproduced by other researchers with the same or a different target and workflows. One must ensure to install all required applications, Taverna and the optimization plugin as described at http://ms-utils.org/Taverna_Optimization.pdf.

## Methods

### Test samples and sequences

In this study, we used six representative datasets from two different organisms and three different types of mass analyzers. Three datasets were generated in our own lab and three fetched from the PRIDE repository [20]. As a prokaryote with a small genome and limited number of modified peptides, we used an *E. coli* whole-cell lysate, prepared as described by Mostovenko *et al*. [21]. This sample was analyzed both by high-resolution TOF mass spectrometer and in an ion trap. As a eukaryote with a larger genome and frequent occurrence of modified peptides, we used a sample of human plasma isolated from blood drawn from a self-declared healthy individual after verbal informed consent according to local guidelines approved by the Medical Ethics Committee at the Leiden University Medical Center. The human plasma sample was analyzed on the same ion trap as the *E. coli* digest. The three additional datasets were downloaded from PRIDE were an orbitrap dataset from a study of label-free absolute proteome quantification methods using *E. coli* [22] (project PXD000283, dataset #29781), an orbitrap dataset from glioma-derived cancer stem cells [23] (PXD000563, file “GSC11_24h_R1.raw”) and a TOF dataset of human induced pluripotent stem cells [24] (PXD000071, “120118ry_201B7-32_2_2.wiff”). These datasets cover three common types of mass analyzers with varying resolving power and mass measurement accuracy as well as organisms with small and large genomes. UniProt reference proteomes data for *E. coli* (April 2013, 4,439 sequences and same number of decoys) and *H. sapiens* (April 2013, 89,601 sequences including isoforms and the same number of decoys) was used for peptide identification using the X!Tandem [25] sequence search engine.

### Liquid chromatography – tandem mass spectrometry

The ion trap only datasets were generated as follows. Two μL of each tryptic digest were loaded and desalted on a 300 μm-i.d. 5-mm PepMap C18 trap column (Dionex, Sunnyvale, CA) and separated by reversed-phase liquid chromatography using a 15-cm, 300 μm-i.d. ChromXP C18 column (Eksigent, Dublin, CA) connected to a splitless NanoLC-Ultra 2D plus system (Eksigent) with a linear 90-min gradient from 4 to 33 % acetonitrile in 0.05 % formic acid and a constant flow rate of 4 μL/min. The LC system was coupled to an amaZon ETD ion trap (Bruker Daltonics, Bremen, Germany) via a CaptiveSpray™ ESI source. After each MS scan, up to 10 abundant multiply charged species in *m*/*z* 300-1300 were selected for MS/MS and excluded for one minute after having been selected twice for MS/MS. Each individual scan or tandem mass spectrum was saved to disk. The LC system was controlled by HyStar 3.2 and the ion trap by trapControl 7.0. To generate a hybrid TOF/ion trap dataset, the *E. coli* digest was loaded and desalted as above, separated on a 15-cm, 75 μm-i.d PepMap C18 column in an Ultimate 3000 LC system (Thermo Scientific, Sunnyvale, CA) with a 180-min 300 nL/min piece-wise linear gradient with the following breakpoints: 2 % B at 0 and 10 min, 5 % B at 25 min, 25 % B at 165 min, 30 % B at 175 min and 35 % B at 190 min, where B is 95 % acetonitrile and 0.1 % formic acid. The LC system was coupled simultaneously to a maXis high-resolution-TOF (also Bruker) and an amaZon speed ion trap using a post-column flow splitter (RePlay™, Advion, Ithaca, NY), both with the CaptiveSpray™ ESI source.

### Optimization of the X!Tandem workflow

**Fig. 1:**
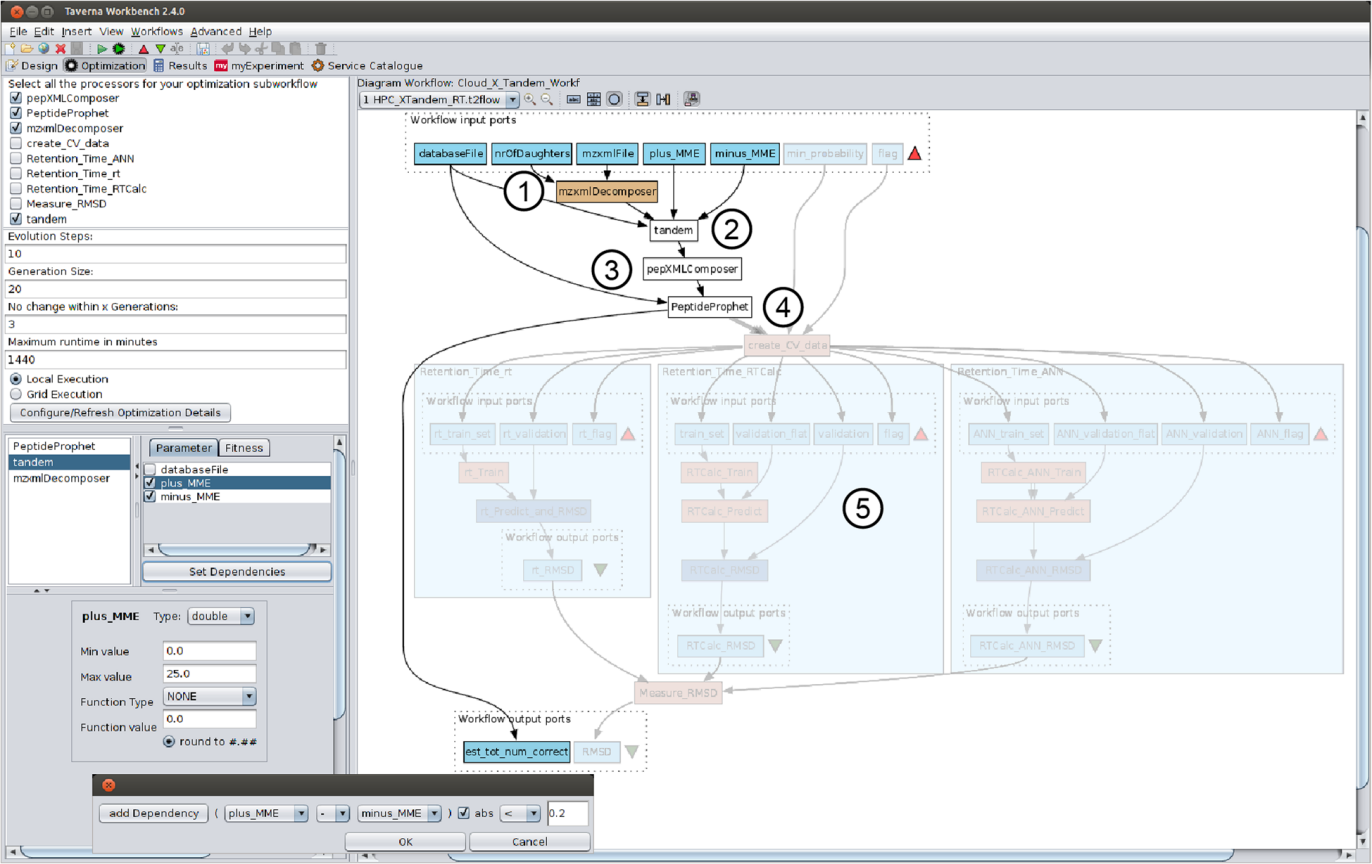
The complete peptide matching and retention time workflow within the optimization perspective. We optimized the workflow in two stages. The figure above shows the optimization of the X!Tandem peptide identification with following major steps: 1. decomposing the input files, 2. database search by X!Tandem, 3. recomposing the output files 4. statistical analysis of the result by PeptideProphet and 5. modeling of chromatographic retention times using an optimum choice of training set and prediction algorithm. The parts of the workflow, here the retention time prediction, are made partially transparent as they were not part of the optimization within this stage. The bottom left window also shows the dependency settings for the two input parameter MME+ and MME

Scientific workflows are becoming more common in large-scale proteomics data analysis [15, 26]. Some of the authors already designed parts of the current use case as scientific workflows within the Taverna workflow management system. These workflows included the decomposition of mass spectrometry data and peptide identification via X!Tandem or SpectraST [27]. The workflows were made highly parallel for an optimal execution in a cloud environment [16]. We extended the X!Tandem workflow and shifted the computationally intensive X!Tandem execution to the Grid using the Taverna UNICORE plugin [28]. The X!Tandem workflow is highlighted in Fig. 1 (The workflow can be downloaded at http://www.myexperiment.org/workflows/3693.html) representing the following major steps: 1. decomposing the input files, 2. database search by X!Tandem, 3. recomposing the output files 4. statistical analysis of the result by PeptideProphet and 5. modeling of chromatographic retention times using an optimum choice of training set and prediction algorithm. This workflow can be used for conventional execution and for the optimization procedure in Taverna. The workflow in Fig. 1 is shown from the optimization perspective, which integrates graphical user interface elements for the optimization specification and run. The perspective is provided by the optimization framework [19] and partly more specifically detailed by the respective optimization method plugin. Figure 1 also illustrates that the workflow does not need any modification for the usage in the optimization framework. The optimization perspective offers different panels that enable the user to: 1. define the sub-workflow for optimization, 2. set optimization-specific values, 3. determine required input parameters, such as MMEs, and the optimization target (which is represented by one output port of the workflow) and 4. specify parameter data types, ranges and dependencies (panels 2, 3 and 4 are provided by the extended plugin). Using all these optimization specification parameter, expert knowledge can be taken into account during the optimization procedure to limit the search space from the outset. Depending on the runtime of one workflow execution and the total runtime of a complete optimization, such limits are often required to make optimization feasible. Additionally, some parameter combinations might be obviously useless, and should be omitted. For the optimization of X!Tandem and PeptideProphet only the highlighted workflow is used.

**Fig. 2:**
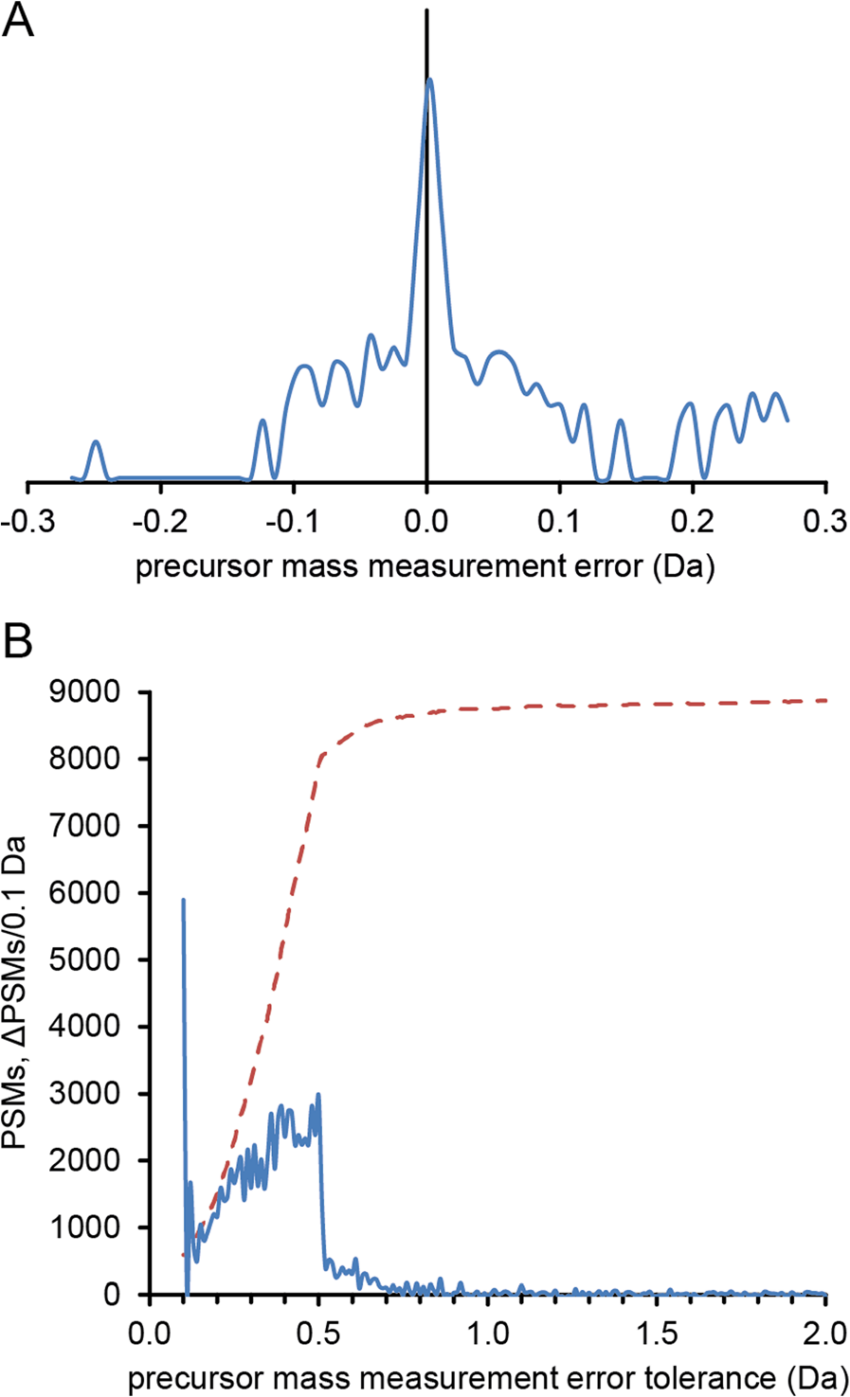
**a** Mass measurement error distribution (density at 1 % PSM-level FDR) in the *E. coli* maXis TOF-amaZon ion trap dataset (logarithmic scale) and (**b**) estimated number of identified correct 2+ spectra as function of MME tolerance in an X!Tandem search, using equal positive and negative MME tolerances and allowing for “isotope error” (dashed, red). When the gaps between the windows centered on each isotope (i.e. 0, 1 and 2 Da) close, the number of 2+ PSMs gained per unit MME (*i.e*. the derivative of the number of PSMs with respect to the MME) drops drastically (blue)

It is reasonable to assume the optimum MME tolerance for peptide-spectrum matching has some relationship with the MME itself (Fig. 2a). An MME tolerance window narrower than the MME distribution will discard many potential peptide-spectrum matches (PSMs), while a much broader window will fail to take advantage of high mass measurement accuracy. The discrimination between correct (true positive) and incorrect (false positive) peptide-spectrum matches decreases the more sequences a spectrum is matched against, as the score of the *best* matching random (false positive) peptide increases with the number of sequences searched. In the workflow optimizations here we allowed both the maximum positive and negative MMEs to vary between 0 and 25 Da for all test datasets. The MMEs are not necessarily symmetric. Often, mass spectrometers are not perfectly calibrated, and a small but significant bias can be found after identifying the peptides. In addition, the instrument sometimes selects an isotopic peak other than the monoisotopic, resulting in a systematic error of +1 or perhaps +2 Da. We therefore allowed the maximum positive and negative MME to be independently varied over the entire range and the “isotope error” in X!Tandem. In this optimization process, strict tryptic enzyme specificity was also assumed, allowing for two missed cleavages. Carbamidomethylation of cysteines was considered a fixed post-translational modification in addition to the variable modifications included by default, such as N-terminal pyroglutamate from glutamine or glutamic acid. As we used the default k-score with the TPP version of X!Tandem, the fragment ion tolerances are not used in the scoring, which is based on a dot product with a fixed bin size.

In X!Tandem and many other search engines, it is possible to define not only a number of allowed missed cleavage sites within a peptide, but also the *fidelity* of the enzyme. The latter allows for zero or one of the peptide termini not conforming to the enzymatic specificity and are in X!Tandem referred to as “full” – strict tryptic cleavage – meaning that both termini have to be the result of tryptic cleavages unless the peptide is from the protein N- or C-terminus, and “semi”, meaning that only one site of the termini has to result from cleavage by trypsin. In software such as Mascot, this is not an independent parameter but implemented as a virtual enzyme (“semiTrypsin”). In order to fully demonstrate the advantage of the optimization framework, we performed a second optimization on the *E. coli* ion trap data, starting from the MME tolerance optimum, with two additional parameters included in the optimization process: the number of missed cleavages (integer ∈ [0, 4]) and the enzymatic fidelity defined by a Boolean representing’full’ (default) or’semi-tryptic’.

There are many methods available for finding the optimum of a given function. One should take care if using a method based on derivatives (numerical, as it is not reasonable to find an analytical expression). For instance, when allowing isotope errors (or “# 13C” in Mascot), the derivative of the number of PSMs as a function of the allowed MME is discontinuous where the sum of the negative and positive error is 1 Da (Fig. 2b). In search engines having only one MME tolerance parameter, *i.e*. the same positive and negative maximum MME, this happens exactly at 0.5 Da maximum MME. This is easy to understand, as the two or three searched mass windows become one, and the window is expanding further along two edges rather than four or six. There are also a number of discrete variables that can be modified and that influence the peptide-spectrum matching, for example isotope error, missed cleavages, minimum and maximum peptide length, and both fixed and variable post-translational modifications. These parameters are often binary (isotope error, included PTMs) but can sometimes take on any integer value in a small range (peptide size, missed cleavages, maximum number of variable PTMs per peptide). Additionally, the choice of search algorithm itself can be subject to optimization. Most database search engines have equivalent parameters, such as MME, missed cleavages, peptide size and considered PTMs. In order to optimize the described parameters above, we use the Taverna workflow optimization framework that employs an evolutionary algorithmto optimize multiple continuous, discrete or binary parameters and find the combination that gives the best global performance according to a user-defined target, or fitness function. Here we use as fitness function the number of estimated correct PSMs from doubly charged precursors given by PeptideProphet using decoys and the non-parametric model divided by the total number of tandem mass spectra or the root-mean-square deviation of predicted peptide retention time, as these are robust and easily calculated metrics. We use the PSMs as they are closer to the data and better represent discrete units of information in a bottom-up proteomics experiment – in quantitation by spectral counting for example – than the perhaps biologically more relevant number of unique peptides or proteome coverage. However, there is no reason to assume that optimizing for the number of PSMs would not also provide good parameters for unique peptides and proteins.

The Taverna optimization framework used in this paper offers a generic application programming interface to extend Taverna with various types of optimization as well as optimization algorithms. For non-linear and partially discrete problems such as algorithms and simulations used in scientific workflows, the fitness landscape may be rugged and not assessable in many places. Properly dealing with these issues requires a robust and versatile method, such as metaheuristic optimization. The intrinsic parallelizability of such methods is a major advantage in large optimization problems such as those addressed here. Evolutionary algorithms are the parallel metaheuristic of preference [29] and thus the optimization pluginwe used in this paper was implemented with Evolutionary Algorithms, in detail Genetic Algorithms (GA) [30]. Additional motivations for using GAs are their simplicity, proven performance, versatility and success in the life sciences [31]. The plugin uses an existing Genetic-Algorithm-library, JGAP [32], and was adapted to workflow parameter optimization by coding each input parameter as a “gene” on a “chromosome”, where each chromosome contains a particular combination of input parameters. In each generation, individual instances of the workflow are executed; one for each chromosome (parameter set). After a user-defined number of generations or other abort criteria, the framework presents the user with the optimal or best parameter set found. Additional statistics, which we will also use in this paper, can be saved after the optimization phase. By using this generic optimization framework and the extended parameter optimization plugin, we obtain a better and more robust parameter set than by using defaults or refining parameters by trial and error. Additionally, there is no need for any prior knowledge about optimization techniques, as the framework and plugin manage all aspects of the optimization. The framework enables researchers to easily optimize scientific workflows and thus increase the scientific output more efficiently than using trial and error or parameter sweeps. More information about the optimization framework, the optimization process and other examples can be found at http://ms-utils.org/Taverna_Optimization.pdf or [33].

All computing intensive executions (e.g. X!Tandem) performed during the optimizations in this work were conducted on a Grid that was set up by the Grid software UNICORE [34]. The calculations were executed on a cluster within the Grid with 206 compute nodes, each of which consists of two 2.66 GHz Intel Xeon 6-core processors and 96 GiB main memory. For the execution on the Grid, 4 CPUs per job were requested by the user. The scheduling and execution of the jobs were handled by UNICORE, as described previously [28].

### Optimization of retention time prediction

**Fig. 3:**
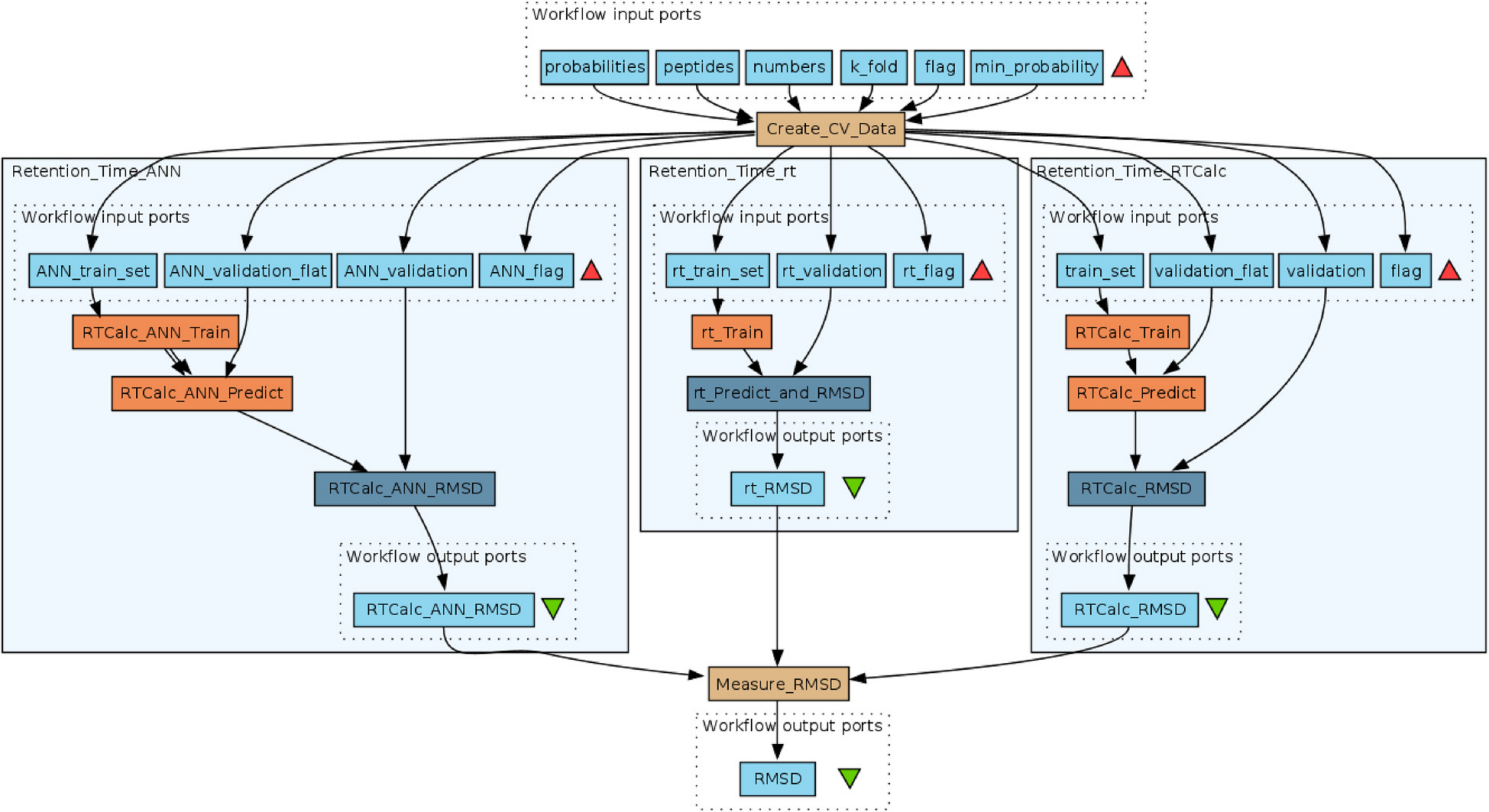
Stand-alone workflow for retention time modeling and prediction. Each of the three embedded subworkflows corresponds to one particular retention time model. The subworkflows can be switched on and off by a flag. In each workflow run, only one of the subworkflows is executed. This flag was used as a parameter in the workflow optimization. Taverna workflows visualize workflow inputs by a red triangle and outputs by a green triangle. This also holds for embedded (sub-)workflows

To illustrate a different type of optimization, comparing not only parameters but also algorithms, we included a retention time prediction in the workflow. The workflow in Fig. 1, shown in grey was used for the optimization. It can be accessed at http://www.myexperiment.org/workflows/3691.html. In addition to peptide-spectrum matching, we may choose to incorporate additional information about the peptides in the identification or removal of false positives. One way to do this is to train a retention time predictor and use this to remove peptides that do not fit the predicted chromatographic behavior from the list of peptide matches [35]. There are a number of algorithms for this purpose, including the original software “rt” [35] and two different algorithms included in the RTCalc utility in TPP: one based on SSRCalc [36] and one based on the artificial neural network (ANN) method by Petritis and co-workers [37]. To demonstrate how a scientific workflow can choose an optimal path for proteomics data analysis, we designed a workflow to balance the quality (FDR or PeptideProphet probability cutoff) of the training set and the prediction model, to find the model that can best predict the retention times of peptides within the same dataset. The rationale is that the simpler retention time predictors have fewer free parameters and will be trained more robustly by smaller training sets than the potentially better but more complex models requiring much more training data. RTCalc has its own hardcoded internal quality checks that generates an error message and aborts rather than produce a poor or overfitted model. We disabled these checks in the RTCalc source code to level the playing field and allow the optimization framework to independently find the right combination of parameters and algorithm. Alternatively, and for increased robustness, the root-mean-square deviation can be set to a very large (or small) value if RTCalc or rt returns an error due to too few peptides or non-convergence to avoid having the genetic algorithm explore regions where no good solutions could be expected. In addition to the choice between the three algorithms, we simultaneously optimized the PSM probability cutoff as calculated by PeptideProphet for the peptides included in the training sets. This workflow is shown as a stand-alone workflow in Fig. 3 and is also embedded in Fig. 1. The quality of the retention time prediction was evaluated as root-mean-square deviation for 10 % of the peptides held back as a validation set, using the remaining 90 % of the peptides to train the model. These 10 % were then chosen at random 10 times so that each peptide was used exactly once for validation. The PSM probability cutoff for the validation set was constant at *p* = 0.99. The result of this optimization was then used in a downstream workflow removing PSM outliers with measured retention time deviating from the predicted retention time by more than a user-defined absolute Z-score, here 2.0.

## Results and discussion

### Results of X!Tandem optimization

**Fig. 4:**
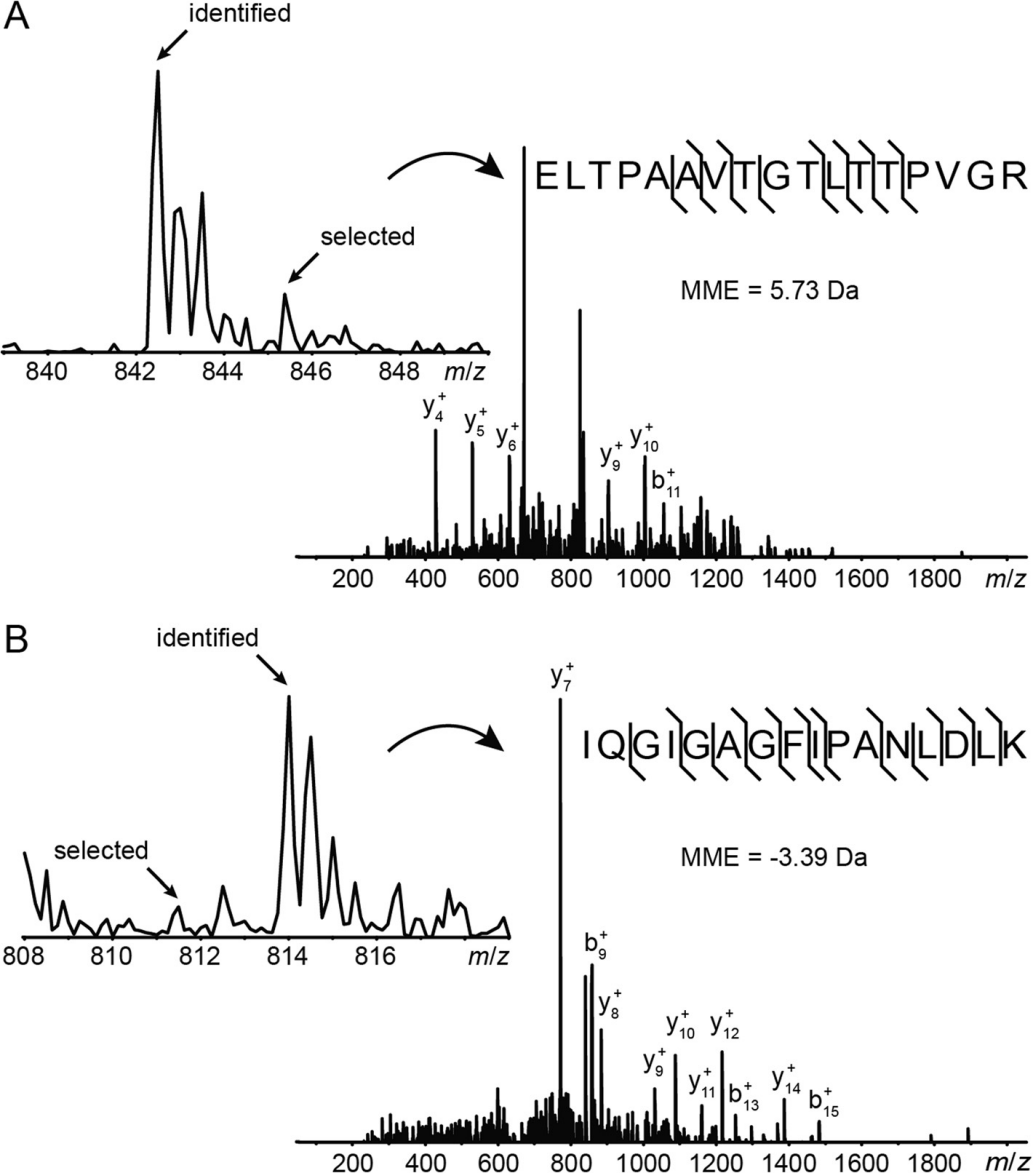
Peptide identifications from non-selected precursors. With larger MME tolerances, here ±5 Da plus isotope error, X!Tandem identified co-eluting peptides with lower (**a**) or higher (**b**) *m*/*z* than the selected precursor but within the precursor isolation window. In A, a peptide with monoisotopic *m*/*z* 842.5 was identified (with PeptideProphet probability cutoff *p* = 0.989) instead of a peptide (or signal) at *m*/*z* 845.3 triggering the MS/MS event. In B, a peptide with *m*/*z* 814.0 is identified (with *p* = 0.992) instead of a peptide at *m*/*z* 811.5. Both precursors were the ninth to be acquired out of ten sorted by intensity for their corresponding MS scans, more than 1.5 s after the MS scans themselves, and both precursors disappear into the background in the subsequent MS scans. These are two examples of almost 100 such PSMs in the *E. coli* ion trap dataset

The optimum MME search windows found for the six test datasets using the ranges for X!Tandem as described above can be found in Tables 1 and 2. Remarkably, in only one of these, the human orbitrap dataset, does the optimum MME search window appear to directly correspond to the mass measurement uncertainty of the instrument (±0.01 Da). The optimum upper MME (MME+) limits for both TOF datasets and the human ion trap dataset as well as the lower MME (MME-) limits for both ion trap datasets were between 5.3 and 9.0 Da. These correspond to 2.6–4.5 *m*/*z* units for a doubly charged peptide and are related to the widths of the precursor ion selection window in the quadrupole or ion trap rather than the mass measurement uncertainty. Already in small MME tolerance windows (<1 Da), one can observe such outliers that cannot be explained by poor instrument performance, but are caused by a second, co-eluting peptide in the same *m*/*z* window as the peptide selected for MS/MS (Fig. 4). The selection window for MS/MS also does not have infinitely sharp boundaries, but allows a fraction of ions through, even if their *m*/*z* is just outside the window as defined in the mass spectrometer control software. This behavior results in a mixed tandem mass spectrum with fragment ions from two or more peptides. When the second, “freeriding” peptide produces more intense fragment ion peaks than the selected peptide, the former peptide is more likely to be identified, as long as it is within the searched mass measurement window. This is especially true when there is no penalty for MMEs, which is the case for Sequest, Mascot, X!Tandem and a number of other common search engines. Although retrospectively making sense, we had not predicted that this effect would dominate the benefit of searching a narrow *m*/*z* window corresponding to the actual MME for all but one of the test datasets.

**Table 1:** Results from the X!Tandem and PeptideProphet optimization of the six test datasets with information on number of unique peptides and the optimal MME

Species	Mass analyzer	PSMs	PSMs [M + 2H]^2+^	Unique	opt. MME-	opt. MME+	opt. PSMs	opt. PSMs [M + 2H]^2+^	opt. unique
*E. coli*	ion trap	12889	8393	1197	0.31	7.32	14057 (+9.1 %)	9260 (+10.3 %)	1296 (+8.3 %)
*E. coli*	TOF	11285	9608	3840	5.57	17.62	13221 (+17.2 %)	11264 (+17.2 %)	4356 (+13.4 %)
*E. coli*	orbitrap	18343	11129	7419	0.80	15.00	18548 (+1.1 %)	11366 (+2.1 %)	7526 (+1.4 %)
*H. sapiens*	ion trap	8152	5316	528	9.02	5.32	8490 (+4.1 %)	5571 (+4.8 %)	577 (+9.3 %)
*H. sapiens*	TOF	8650	5802	3835	0.31	6.33	8619 (-0.4 %)	5833 (+0.5 %)	4300 (+12.1 %)
*H. sapiens*	orbitrap	17413	12239	4287	0.01	0.01	19551 (+12.3 %)	13772 (+12.5 %)	5164 (+20.5 %)

**Table 2:** Results from the X!Tandem and PeptideProphet optimization of the six test datasets with information on execution times and the total time for the optimization

Dataset	Runtime (def.)	Runtime (opt.)	Runtime (max)	Optimization time
*E. coli* (ion trap)	00:01:17	00:07:11	00:34:03	04:58:32
*E. coli* (TOF)	00:06:42	00:09:56	00:14:03	04:07:15
*E. coli* (orbitrap)	00:08:08	00:08:30	00:09:45	03:09:40
*H. sapiens* (ion trap)	00:19:34	03:06:00	09:17:00	29:45:13
*H. sapiens* (TOF)	00:13:06	00:52:13	03:10:00	23:07:18
*H. sapiens* (orbitrap)	00:04:22	00:09:05	02:40:54	12:36:58

**Fig. 5:**
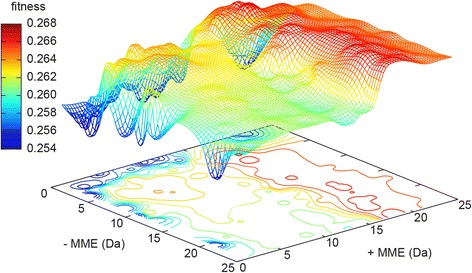
Optimization of MME window for X!Tandem on the hybrid ion trap/maXis dataset described above, with fitness defined at the number of correctly identified spectra from doubly charged precursor divided by the total number of tandem mass spectra. The surface was interpolated and visualized outside Taverna using gnuplot with the dgrid3d and countour base functions, although similar graphics could in the future possibly also be created in an Rshell *inside* the Taverna workflow. The clearly visible ridge between 16 and 21 Da positive MME corresponds to the pyroglutamate/ammonia loss resonance adding 437 peptide-spectrum matches (4 % of all PSMs) with 1 % FDR and MMEs 15-20 Da, nearly all by assigning actual NH_3_-loss *b*-ions as regular *b*-ions with 17 Da MME. As comparison, there are only 9 PSMs in the MME window between 10 and 15 Da. Similar ridges are seen in at least four of the six datasets (supplemental information), although the global optimum is not always found along this ridge. It should be noted that the standard error in the actual mass measurement is below 2 ppm in this dataset, but that this number has very little relevance for the optimum MME window for X!Tandem in a search of this dataset with only one variable modification and in a small sequence database

In all *E. coli* datasets we gain a number of these peptide-spectrum matches when we allow MMEs of 5 Da compared to 0.5 Da (1,759 additional PSMs in the ion trap dataset and 2,032 in the hybrid maXis/ion trap dataset). Naïvely, one may assume that outside this ~5 Da window, no more true PSMs will be found by stretching the search window further. However, this is not the case. When expanding the search to allow MMEs of up to 15 Da or more (plus isotope error of 1 or 2 Da), a number of new PSMs appear. These are caused by the matching of theoretical spectra from peptides with N-terminal pyroglutamate from glutamine or glutamic acid with measured spectra of unmodified peptides experiencing ammonia or water loss from the N-terminal glutamine/glutamic acid during fragmentation. This shifts the *b*-ions by 17 (NH_3_) or 18 (H_2_O), indistinguishable from the difference between glutamine/glutamic acid and pyroglutamate. As these are now identified as *regular b*-ions rather than *b*-ions with ammonia (b*) or water (*b*^o^) loss, the peptide-spectrum match receives a higher k-score by X!Tandem, bringing a number of these PSMs from just below to above the 1 % FDR threshold, leading to a larger number of correct PSMs (Fig. 5). Of course, a scoring scheme that gives equal weight to *b-*, b*- and *b*^o^-ions and weighs in the MME would still identify the (fully) correct peptide. However, this is not the case in most common search engines that allow arbitrarily large MMEs in the search. This phenomenon shifts the global MME+ optimum to 17.6 Da and 15.0 Da for the ion trap and orbitrap *E. coli* datasets respectively (Table 1) and produce local optima along a ridge with MME+ 15–18 Da in the other datasets. In the orbitrap *E. coli* dataset searched with ±25 Da MME tolerance and filtered for 1 % FDR by PeptideProphet, there were also 371 PSMs with MME 15.98–16.02, 300 of which contained at least one methionine and 27 more at least one histidine or tryptophan. There were 22 PSMs with MME 16.98-17.02, 11 of which contained an N-terminal glutamine, 483 PSMs with MME 17.98–18.02, 478 with N-terminal glutamic acid and 3 with N-terminal glutamine, and 8 PSMs with MME 18.98–19.02, out of which 7 contained an N-terminal glutamic acid. In total, 1,572 out of 16,668 PSMs in this search were found outside the [-0.02, 2.02] MME window. Similar patterns were observed for the other datasets. Extending the MME tolerance to ±25 Da actually identifies more spectra (albeit the difference is very small, 16,668 compared to 16,654) than when searching the same dataset with X!Tandem with the ±5 ppm MME tolerance (still allowing isotope error) used in the originally published analysis of the dataset [22].

As we make the mass error tolerance window larger, we also retrieve more random, or false, peptides. The score for the best matching random peptide increases monotonously as a function of MME. In PeptideProphet, this corresponds to a translation of the negative distribution to higher discriminant scores while the positive distribution remains unchanged. At some point, the cost of allowing better random matches will exceed the gain of additional PSMs. In addition, searching a larger window is more computationally expensive, scaling roughly linearly with the width of the error tolerance window. The X!Tandem run time at the optimum varied from 7 to 10 min for the *E. coli* datasets and from 9 min to 3 h for the human datasets (Table 2). Execution time and computational cost were not explicitly considered in the optimizations, and for datasets such as the human TOF data used here, the relatively marginal improvement of 2.5 % additional PSMs may not motivate the 2.5 h additional computational time, though all computationally intensive components of these workflows have been parallelized and can be run on clouds, grids or supercomputers [16]. As mentioned above, it is generally recommended to run these database searches in parallel. When considering the optimization runtime, the entire computational cost consists of the sum of each workflow run. The real runtime of an optimization process is therefore the sum of the longest workflow execution within each generation. For example, if in generation 1 the longest workflow execution took 10 min and in the second generation 12 min, the total time for this optimization was 22 min, with 40 workflows having been executed in these two generations. This is feasible due to the parallel execution mechanism implemented within the optimization framework in Taverna. In any case, the researcher should be aware of the required total compute resources needed for the execution of the workflows. Table 2 also lists the runtimes of the workflow using the default MME tolerances (±0.5 Da), the maximum tolerances (±25 Da) and the optimum window. The times required for the entire optimizations are also included, although the optimization should only be required once for each combination of sample type, instrument and method parameters. Additionally, the time required to perform the specific optimization is given. Again, the researcher should be aware that the actual times may be dependent on the availability of the computing resources and the queuing time.

The results from the second optimization including missed cleavage sites and enzyme fidelity are shown in Table 3. It is clear that allowing only one missed cleavage is slightly better than allowing two missed cleavages and that the default value for the specificity (fully tryptic) was also the best value. The optimum MME tolerances did not change dramatically. The improvement in fitness (fraction of identified 2+ spectra) was less than 1 %, suggesting the initial values were sensibly chosen. Additional parameters can easily be included in the optimization process.

**Table 3:** Results from the second optimization, in which different numbers of missed cleavages and different enzyme fidelities were also investigated for the *E. coli* hybrid ion trap/TOF data

	PSMs	PSMs [M + 2H]^2+^	Unique	MME-	MME+	Missed	Fidelity
Default	14057 (+1.2 %)	9260	1292 (-1.9 %)	0.31	7.32	2	full
Optimized	13888 (-1.2 %)	9282 (+0.2 %)	1271 (-1.9 %)	0.28	7.17	1	full

### Results of retention time prediction optimization

**Fig. 6:**
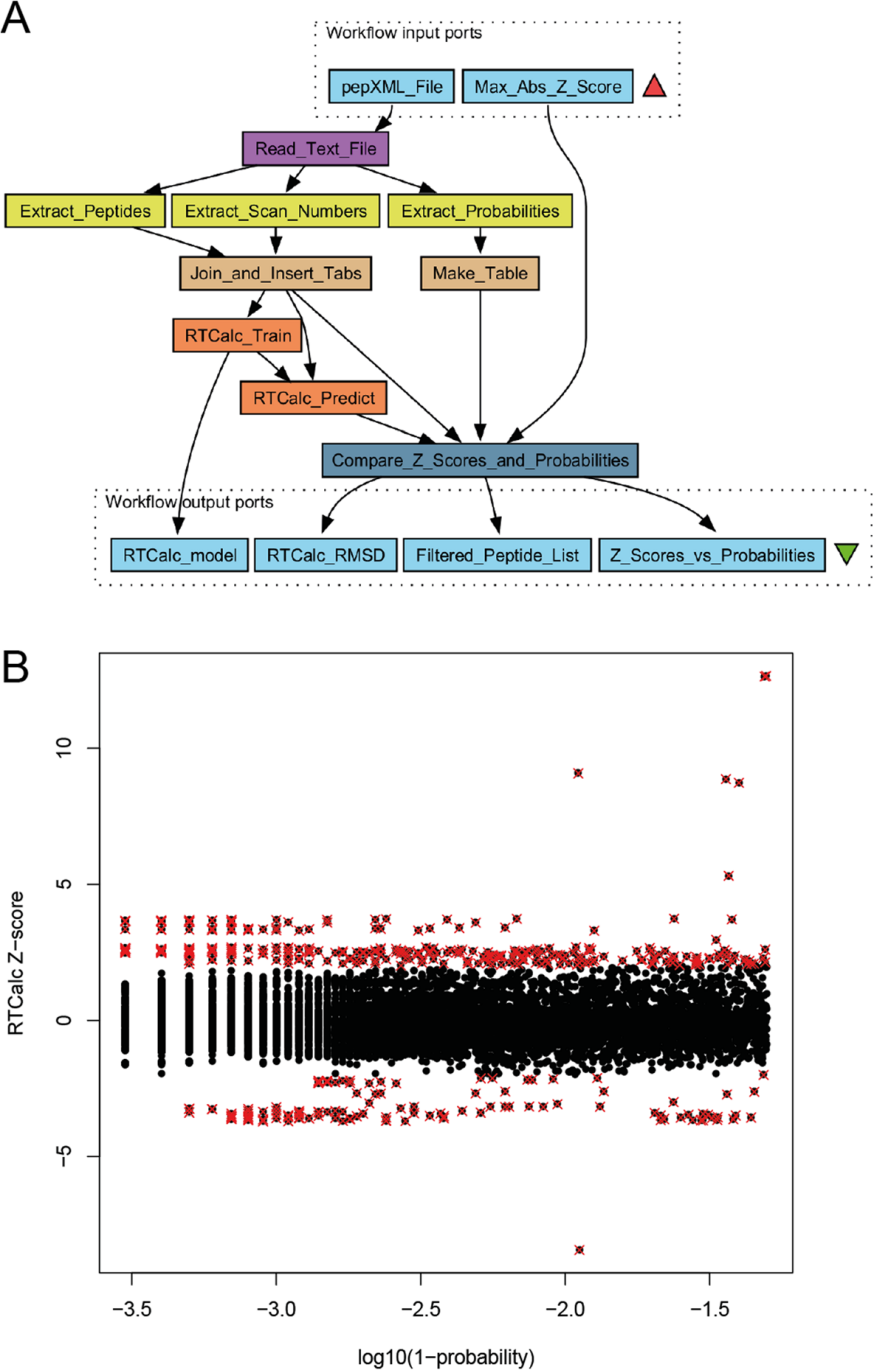
Workflow using the best retention time predictor (SSRCalc in RTCalc) to filter a list of PSMs based on the agreement between measured and predicted retention time, assuming the identification is correct (**a**). The user selects a Z-score threshold to remove outliers, which are likely due to false identifications. Here we used a maximum absolute Z-score of 2 to demonstrate the workflow, although this may be overly conservative, as a few PSMs of very high PeptideProphet probability are also removed (**b**). The workflow is available on myExperiment (workflow #4042)

As expected, the number of peptides in the training sets used here were not sufficient to produce an accurate model using the artificial neural network algorithm. When there were more than ca. 70 peptides in the training set, the RTCalc coefficient (SSRCalc) model performed best. When there were between 52 and 70 peptides, rt performed better, and for 21–52 peptides in the training set, only rt produced a model at all. No model was returned when having 21 peptides or fewer in the training set. In absence of quality checks, the minimum number of peptides required to produce a model is solely determined by the number of free parameters (terms) in the model. It is possible that for very large training sets (>100,000 peptides), the ANN model will outperform the SSRCalc-derived model in RTCalc [37]. The optimum algorithm, SSRCalc, was then selected for use in a new workflow (Fig. 6a). A few outliers could be removed from the *E. coli* ion trap X!Tandem results with PeptideProphet *p* ≥ 0.95 and maximum absolute Z-score of 2, the most conspicuous having probabilities *p* < 0.99 or *log*_10_(1 - *p*) > -2 of being correct in the first place (Fig. 6b).

## Discussion

The two examples shown here demonstrate that systematic exploration of parameters and algorithms for data analysis in mass spectrometry based proteomics can achieve at least two things. First and foremost, re-evaluating legacy parameter and model choices allows more peptides and proteins to be identified, which may allow more biologically relevant information to be extracted from the raw mass spectrometry data. The optimization should be done on a representative dataset, or a fraction of all the spectra, for instance sampled using random data decomposition [16]. Secondly, exploring different combinations of parameters and algorithms leads to new insight into the data and the algorithms themselves – for example the ammonia loss *b*-ion spectra identified as peptides with N-terminal pyroglutamate and the behavior of the retention time predictors for different size training sets. These phenomena were not chosen for investigation, but uncovered during the parameter optimization when allowing the parameters to vary over a wide range. The optimum MME windows were found to be asymmetric with a larger tolerance of positive MMEs. In one dataset, the optimal positive MME was found along the ridge (+17.62 Da) corresponding to the pyroglutamate/NH_3_-loss PSMs. The other optimal MMEs were found either just outside the actual mass measurement errors (-0.31 or -0.50 Da) or just outside the MME corresponding to the precursor isolation window as illustrated in Fig. 4 (-5.57, +7.32 or +7.95 Da). Similar observations were independently reported by three different groups at a recent international conference [38–40], including data from a Q Exactive Orbitrap [41]. The phenomenon makes perfect sense given the distribution of MMEs observed when allowing very large MMEs in the X!Tandem search, with few PSMs with MMEs below -6 or between 8 and 15 Da. An important point here is that the genetic algorithm searches a very large parameter space, and would also be able to find an optimum very close to zero if one exists for very accurate precursor mass measurements.

It is also important to be aware of a number of effects that can mislead optimization procedures such as the ones followed here. For some combination of parameters, possibly very far from optimal, the PeptideProphet expectation-maximization (EM) may fail to find the globally best fit to the measured discriminant score distribution. This can sometimes be explained by a noisy discriminant score distribution, but sometimes the PeptideProphet EM algorithm gets stuck in a local minimum. We therefore settled for the target/decoy and the non-parametric model of “2+” spectra in PeptideProphet, as this does not fail over the range of parameters investigated in this study, whereas it occasionally fails for “1+” and “3+” spectra, especially when using the parametric model. The optimum found should still be a very good parameter choice for slightly different targets, as roughly two thirds of the identifiable spectra are from doubly charged precursors. The workflow feedback in the form of parameter surfaces is helpful in visually validating the optimization, and catching numbers returned from a failed EM that are obviously erroneous (such as identifying nearly 100 % of the spectra). Over smaller ranges and for more or better data and algorithms, the parametric model may still function sufficiently well for use in optimization. A different optimization target, such as the number of unique identified peptides, may theoretically produce a smaller optimum MME tolerance, as many of the peptides identified in the larger windows, such as the co-eluting peptides in Fig. 4, would have also been selected for MS/MS and identified from different spectra in the same dataset. However, it is good to remember that random (false) matches tend to be to unique peptides, and that optimizing for the number of unique peptides or proteins will have a positive bias toward spurious identifications.

The usage of the Taverna workflow management system and the optimization framework produced only a small overhead in this experiment. Even if scientific workflows are still new in the proteomics field [15], many researchers are already familiar with the usage of scientific workflow management systems like Taverna. As Taverna is implemented in Java, it can be executed as a Java application without installation and thus typically on every machine. With the Taverna graphical interface, users can design their own workflows or reuse existing ones from a repository [42]. Some workflows require access to or installation of applications that will be called by the workflow. Adaptation is sometimes needed in order to run the workflows on one’s own machine. This procedure is very dynamic in Taverna and cannot be described in general. References and further literature can be found at http://www.taverna.org.uk. The workflow optimization plugin is designed as a standard Taverna plugin and can be installed automatically by adding the download page to Taverna (as described in http://ms-utils.org/Taverna_Optimization.pdf). To enable the optimization process on a workflow, a graphical user interface is offered to select the sub-workflow, define termination criteria, and specify parameters, along with their ranges and dependencies. A modification of the workflow is not required for the optimization. After the optimization process, the result is presented to the user, who can store the entire optimization process including execution statistics and other information. For more detailed information on the optimization plugin, please refer to [33].

## Conclusion

We used a new optimization framework to optimize a scientific workflow for peptide-spectrum matching and retention time prediction. The two steps were optimized separately from each other in the Taverna Workflow Manager. With the optimization framework users can optimize various parameters of any algorithm or tool within a scientific workflow. In our use case we allowed a much larger MME window for X!Tandem than typically used. With this setup we had been able to find new PSMs outside of the commonly searched MME window. These PSMs were primarily due to the unpredicted matching of spectra from peptides with N-terminal pyroglutamate from glutamine or glutamic acid with measured spectra of unmodified peptides experiencing ammonia or water loss from the N-terminal glutamine/glutamic acid during fragmentation.

In conclusion, we suggest an open mind and perhaps a more widely open search window is needed whenever looking at data from new types of experiments or new mass spectrometers. Scientific workflows, for example in Taverna, have many advantages for analysis of large proteomics datasets, such as comprehension, shareability, provenance, interfacing with cloud or grid computing. In combination with the Taverna optimization framework, the workflow can then be optimized with respect to parameters as well as algorithms, on-the-fly and fully transparently. Additional search parameters and exclusion criteria, such as minimum number of peaks, minimum fragment *m*/*z* and minimum peptide length, may also deserve investigation, although short peptides tend to less protein-specific and therefore of less value in practice.

### Availability of supporting data

All software and workflows are freely available at http://unicore-dev.zam.kfa-juelich.de/taverna/plugins/ and from myExperiment.org. The installation and usage guide is available at http://ms-utils.org/Taverna_Optimization.pdf. At http://www.myexperiment.org/workflows/3693.html the X!Tandem and PeptideProphet workflow is available. The workflow for the retention time prediction optimization can be accessed at http://www.myexperiment.org/workflows/3691.html. The liquid chromatography-tandem mass spectrometry datasets produced in-house, including the hybrid ion trap/maXis data, are available from http://cpm.lumc.nl/export/public_datasets/.
